# Potential Help-Seeking Behaviors Associated with Better Self-Rated Health among Rural Older Patients: A Cross-Sectional Study

**DOI:** 10.3390/ijerph18179116

**Published:** 2021-08-29

**Authors:** Ryuichi Ohta, Mikiya Sato, Jun Kitayuguchi, Tetsuhiro Maeno, Chiaki Sano

**Affiliations:** 1 Community Care, Unnan City Hospital, Unnan 699-1221, Japan; 2 Department of Primary Care and Medical Education, Graduate School of Comprehensive Human Sciences, University of Tsukuba, Tsukuba 305-8575, Japan; 3 Faculty of Medicine, University of Tsukuba, Tsukuba 305-8575, Japan; mikiya.sato@shi-g.com (M.S.); maenote@md.tsukuba.ac.jp (T.M.); 4 Health Services Center, Occupational Safety and Health Department, Human Resources Group, Sumitomo Heavy Industries, Ltd., Tokyo 141-6025, Japan; 5 Physical Education and Medicine Research Center Unnan, Unnan 699-1105, Japan; junk_907@yahoo.co.jp; 6 Department of Community Medicine Management, Faculty of Medicine, Shimane University, 89-1 Enya cho, Izumo 693-8501, Japan; sanochi@med.shimane-u.ac.jp

**Keywords:** help-seeking behavior, self-rated health, rural older people, Japan, health education

## Abstract

Help-seeking behaviors (HSB) for mild symptoms vary because of differences in health care resources and patients’ backgrounds. Potential HSBs for lay and professional care use are related to patients’ health conditions. However, there is a lack of evidence of the relation between them. This study examined the relation between patients’ potential HSBs and self-rated health (SRH). The cross-sectional study involved 169 patients, aged above 65 years, who visited a Japanese rural clinic. A validated checklist was used to assess potential patients’ HSBs. A chi-square test and logistic regression were performed to examine the relation between patients’ self-rated health and HSB regarding lay and professional care use. Participants were 77.5 years old, on average (SD = 8.3). Results reveal that having regular exercise habits (OR = 2.42, *p* = 0.04), adequate sleep (OR = 4.35, *p* = 0.006), work (OR = 2.59, *p* = 0.03), high socioeconomic status (OR = 6.67, *p* = 0.001), and using both lay and professional care (OR = 2.39, *p* = 0.046) were significantly correlated with high self-rated health. Living alone was negatively correlated with higher SRH (OR = 0.23, *p* = 0.015). To improve rural patients’ health care, in addition to improving their health management skills, potential HSB for mild symptoms should be investigated and interventions that consider patients’ socioeconomic factors and living conditions should be implemented.

## 1. Introduction

Help-seeking behavior (HSB) is the human behavior of keeping healthy and seeking treatment for symptoms [1]. Because it is essential for maintaining one’s health, everyone should have appropriate HSB for his/her health [2]. Having appropriate HSB necessitates a balance between lay and professional care [3]. Lay care refers to care provided by lay people who have received no formal training and are not paid, such as self-care and care by relatives, friends, and self-help groups [4]. Professional care refers to care provided by trained and paid professionals, usually in a formal setting [4]. The efficient use of lay and professional care can reduce inappropriate HSBs for treating mild symptoms [5]. A reduction in inappropriate HSBs in response to mild symptoms can encourage people to use medical care more efficiently and reduce over-prescriptions and the rate of polypharmacy among older patients with multimorbidity.

HSBs vary because of differences in health care resources and patient demographics, such as socioeconomic factors and cultural backgrounds [2,6]. Citizens in urban areas have access to ample medical resources and can choose from and approach various medical and care professionals [7]. In contrast, citizens in rural areas may lack sufficient resources for medical care, which may lead to inappropriate HSB [8]. Rural residents have exhibited various HSBs for mild and severe symptoms, some of which may lead to severe problems, such as delayed treatment of cardiac and brain infarction [5,9]. Furthermore, they may lack information on medical issues regarding isolation and access to healthcare information [10,11]. Older adults, particularly those in rural areas, collect information from television or newspapers [12], which indicates a lack of access to information and makes them distinct from younger generations [13,14]. Rural older patients lacking proper standards to act on their symptoms and anxiety may practice inappropriate HSB, such as excessive use of medical resources for mild symptoms, which, in turn, may lead to undesirable consequences for their health [15,16]. Therefore, the HSB in rural older people should be facilitated by utilizing various interventions from multiple perspectives [17]. As rural settings may limit older people’s HSB, they can mainly use the support of primary care physicians for their mild symptoms, with fewer instances of self-medication and help from community members. Moreover, they may not be able to effectively use healthcare resources from their communities.

The clarification of the correlation existing between HSBs and health conditions can motivate rural older people to alter their HSBs for mild symptoms. Thus, various studies have emphasized the appropriate use of healthcare resources from the perspectives of healthcare costs and exhaustion of healthcare professionals [6,7,8,9]. However, to the best of our knowledge, there is no research showing that potential HSB for mild symptoms can be associated with patients’ health conditions, such as self-rated health (SRH), one of the self-reported outcomes among rural older patients. SRH contributes to people’s quality of life; better SRH should be attained from better healthcare. Therefore, our research question is, “Is there a positive relationship between SRH and potential HSB in response to mild symptoms?” Older people may feel unmet needs for healthcare [18,19]. Therefore, a better understanding of the relationship between SRH and potential HSB in response to mild symptoms may lead to the development of appropriate interventions to facilitate HSBs for mild symptoms for the satisfaction of older patients’ needs. Older adult patients in rural areas can be motivated to provide the appropriate HSB for their health even in situations with fewer healthcare resources and lay care. However, no research has clarified the relationship between health outcomes and potential HSBs. Therefore, this research aimed to investigate the relationship between SRH and potential HSBs of older adult patients in rural areas.

## 2. Methods

### 2.1. Setting

Most of Unnan City in the southeast of Shimane Prefecture, Japan, is covered with forest. It is one of the most rural cities in Japan. The results of a 2017 survey estimated the city’s population at 38,882 (18,720 males and 20,162 females), with an aging rate of 37.82%, which was projected to reach 50% by 2025 [20]. Each family tends to live separately. Kakeya and Tai Clinic are rural clinics in Kakeya and Yoshida Town, situated in the most northern part of Unnan City. Both clinics are approximately 30 km from Unnan City Hospital, the only general hospital in the city. At the time of this study, Kakeya Clinic had five registered physicians, two nurses, and no admission facilities. The Tai Clinic had three registered physicians, two nurses, and no admission facilities. All physicians in both clinics were family medicine specialists.

### 2.2. Participants 

Participants were patients aged over 65 years who regularly visited Kakeya and Tai clinics. All lived in the Kakeya or Tai district. They were informed about this research by posting the content of this research in the clinics. The inclusion criteria were as follows: being over 65 years old, regularly visiting clinics or hospitals, and being able to read, write, and hear properly. Patients who could not read, write, or hear well enough to answer the instrument, including those with dementia and cognitive impairment, were excluded.

### 2.3. Measurements 

Questionnaires were administered to all participants and collected by the clinic’s nurses. The measures were completed at the clinic’s waiting room. To measure patients’ potential HSB for acute mild symptoms in their everyday lives, a validated question regarding the choice of lay or professional care was used for their mild symptoms. The choice of lay care included doing nothing, self-care (enduring it, sleeping, resting, or taking a bath), seeking information, consulting family and friends, consulting community members, using complementary medicine, using home medicine, and buying over-the-counter drugs. The choice of professional care included consulting pharmacists, consulting primary care physicians, visiting medical institutions (other than primary care physicians), and visiting the emergency rooms of general hospitals (including calling an ambulance) [21]. The following background information was included: age, sex, work conditions, exercise habits, eating habits, sleeping habits, smoking, habitual alcohol drinking, educational levels, living conditions, SRH [22], social support [23], and social capital (regarding whether they could completely rely on neighbors in communities, using a 10-point Likert scale) [24], socioeconomic status (SES), and the 14-item Health Literacy Scale for Japanese Adults (HLS-14) [25]. Based on kinds of diseases, a Charlson Comorbidity Index (CCI) score was calculated for each participant to assess their severity of medical conditions. This index measures the severity of patients’ medical conditions as it relates to the possibility of admissions and mortality [26].

### 2.4. Statistical Analysis 

The results of the question of potential HSB were categorized as lay care, professional care, and use of both forms of care. The other independent variables were categorized dichotomously: age (≥75 = 1, <75 = 0), sex (male = 1, female = 0), work (employed = 1, not employed =0), having exercise habits (more than three times a week = 1, none = 0), balanced eating habits (yes = 1, no = 0), healthy sleeping habits (6 h of sleep a day = 1, no = 0), smoking (yes = 1, no = 0), habitual alcohol drinking (yes = 1, no = 0), educational level (above high school diploma = 1, no = 0), living condition (living with family = 1, living alone = 0), SRH (good or relatively good = 1, relatively bad or bad = 0), social support (having or relatively having = 1, not having or not relatively having = 0), social capital (high (10 to 6) = 1, low (5 to 1) = 0), and SES (high (rich, relatively rich, or not poor) = 1, low (relatively poor or poor) = 0). CCI was dichotomized: CCI ≧ 5 and <5 [26]. Regarding the HLS-14, participants were divided into two groups according to their total HLS-14, above or below the median. In this study, the median of the score of HLS-14 was 49. A univariate and multivariate logistic regression model was used to assess the relationship between potential HSBs and their SRH, with the potential confounding factors of age, sex, living conditions, work, exercise habits, healthy eating habits, healthy sleeping habits, smoking (yes or no), habitual alcohol drinking, educational levels, living conditions, social support, social capital, SES, and health literacy.

### 2.5. Ethical Consideration 

Participants were informed that the data collected in this study would be used for research purposes only. They were also told about the aims of this research, how data would be disclosed, and how their personal information would be protected. Consent was obtained. The study was conducted in accordance with the principles of the Declaration of Helsinki. This study was approved by the Unnan City Hospital Clinical Ethics Committee (approval number: 20190009).

## 3. Results

### 3.1. Participants’ Demographic Data 

The total number of participants was 169. Table 1 shows their demographic information. Their average age was 77.5 years (SD = 8.3), and 45.5% of the participants were male. A total of 41% of the participants had regular exercising habits and reported exercising three times a week; 83.4% had balanced healthy eating habits; 82% had adequate sleep of more than 6 h a day; 44.9% had part-time or regular jobs; 13% had a smoking habit; 33% would habitually drink; 47.3% had an educational background that was above high school level; 60.9% felt that their health was good or relatively good; 88.7% felt that they could have social support from others; 12.4% lived alone; 53.3% felt that they could rely on others in their communities; and 50.9% had high health literacy. Regarding potential HSBs, 15.4% of the participants used lay care only; 27.8% used professional care only; and 56.8% used both types of care. A total of 50.9% of participants had a CCI score of <5. Regarding their health condition, participants had hypertension (92.9%), dyslipidemia (79.9%), and diabetes mellitus (16.0%).

### 3.2. Relation between SRH and Potential HSBs and Other Factors

Based on the univariate analysis, having regular exercise habits (crude odds ratio (COR) = 2.17, *p* = 0.02), a balanced diet (COR = 2.43, *p* = 0.04), adequate sleep (COR = 5.22, *p* < 0.001), work (COR = 1.98, *p* = 0.04), smoking habit (COR = 0.39, *p* = 0.04), high SES (COR = 3.38, *p* = 0.004), and high social capital (COR = 2.53, *p* = 0.004) were significantly correlated with high SRH. Based on the multivariate logistic regression model, having regular exercise habits (adjusted odds ratio (AOR) = 2.42, *p* = 0.04), adequate sleep (AOR = 4.35, *p* = 0.006), work (AOR = 2.59, *p* = 0.03), high SES (AOR = 6.67, *p* = 0.001), and using both lay and professional care (AOR = 2.39, *p* = 0.046) were significantly correlated with high SRH. In contrast, living along was negatively correlated with higher SRH (AOR = 0.23, *p* = 0.015) (Table 2).

## 4. Discussion

This study revealed a positive relationship between SRH and patients’ use of both lay and professional care. The relation was not affected by patients’ SES, living conditions, and disease severity. The appropriate use of care—that is, using both lay and professional care—to fit patients’ symptoms can be related to their health outcomes. This relationship should be followed up, which can lead to the specification of causal relationships.

The positive relation between SRH and the use of both lay and professional care can contribute to the development of educational interventions for patients’ HSB management. Previous studies have suggested that the usage of primary care and emergency medicine may be related to negative impacts on healthcare, which should be revisited [27,28]. However, this study shows the positive relationship between SRH and the usage of both lay and professional care, which can be used to improve rural older patients’ HSB by using limited resources in rural communities. For example, patients must understand when they should use lay or professional care to efficiently access health care resources [29,30]. In this process, they may be forced to modify their original HSB [31]. Their modification can cause mental stress and distress, which may contribute to poor health conditions [30]. However, based on this study’s results, the potential HSB of using both lay and professional care can be correlated with better health outcomes, as SRH can be correlated with quality of life and better healthy life expectancy [32]. Furthermore, the previous research suggested that self-management can be associated with better quality of life [33]. Therefore, this result may be one of the reasons interventions regarding HSB are essential. In addition, rural older patients can depend on professional care and be empowered to use lay care based on the support by communities and healthcare workers.

HSB management can be supported by educational interventions for all patients. In this study, the relation between high SRH and the use of both types of care was prominent after adjusting for the effects of age, sex, SES, living conditions, and disease severity. Each patient can have various approaches to their symptoms. These approaches depend on their surroundings [34]. Although the concrete process of HSB varies, if they follow the potential HSB of using both types of care that fit their symptoms, their health conditions can be improved [35]. A previous review shows that the usage of primary care and emergency medicine can be related to patients’ perspectives of being ill and their experiences in healthcare, which can be affected by patients’ and regional medical contexts [36]. For the moderation of HSB, these sensitive factors should be respected for the establishment of interventions for HSBs. There is insufficient evidence regarding the relationship between HSB, patients’ perceptions, and health outcomes. To investigate the factors related to HSBs in each context, observational and interventional studies should inspect each district and country. 

Educational interventions for HSBs can be adjusted to each community’s conditions and perceptions of people regarding HSBs. Each community has different health care resources and contexts of medicine [37]. Through the investigation of healthcare needs among communities and the relationship between the need for and available resources of healthcare, the interventions can be realistic and valid [38]. In addition, social norms and culture can affect people’s HSBs, which should be respected for the development of educational interventions for HSBs [39,40]. In the development process, various stakeholders should be involved, as the empowerment of citizens and their self-driven interventions are shown to be effective and sustainable in health promotion [41]. Interventional studies regarding HSBs can be performed by examining citizens’ perceptions and motivations. Their perceptions and motivations can be investigated through a qualitative inquiry during interviews and focus groups.

For subsequent studies, the limitations of this study should be strengthened. One of the limitations was the inclusion of participants from a group of patients. All older patients at the clinic were included to improve validity. To improve the external validity of the relationship between the SRH and the use of healthcare resources, the selection of participants was broadened to include citizens from communities. Another limitation was a small number of patients aged ≧ 65 years with various severity of conditions from rural Japanese communities. The CCI may not clarify the severity in patients from outpatient departments. New studies can include more participants affected by different diseases from both urban and rural regions in other countries to examine whether there may be different relationship between SRH and patients’ backgrounds. Furthermore, this study only showed the correlation between SRH and potential HSBs. Thus, future research should attempt to design observational and interventional studies to determine causal relations.

## 5. Conclusions

The potential HSB of using both lay and professional care can be associated with high SRH. An appropriate use of healthcare resources may be associated with better health outcomes. To improve rural patients’ health care, in addition to improving their health management skills, potential HSBs for mild symptoms should be investigated and interventions that consider their SES and living conditions should be implemented. Education on HSB for mild symptoms regarding the use of both lay and professional care can be adjusted for citizens in rural areas, and such HSBs should be further encouraged.

## Figures and Tables

**Table 1:** Participants’ demographic data.

Variable	N = 169
Age, years, mean (SD)	77.5 (8.3)
Sex, male participants %	45.5
Exercise, more than three times a week, yes %	41
Balanced diet, yes %	83.4
Adequate sleep, more than 6 h of sleep a day yes %	82
Work, yes %	44.9
Smoking, yes %	13
Habitual alcohol drinking, yes %	33
High education, above high school diploma, yes %	47.3
SRH, high (good or relatively good) %	60.9
Social support, high %	88.7
Socioeconomic status, high %	82.2
Living alone, %	12.4
Social capital, high %	53.3
Health literacy, more than 49, high %	50.9
Healthcare-seeking behavior, number (%)	
Lay care use only	26 (15.4)
Professional care use only	47 (27.8)
Both care use	96 (56.8)
Charlson comorbidity index, number (%)	
Score = 2	24 (14.2)
Score = 3	22 (13.0)
Score = 4	40 (23.7)
Score = 5	42 (24.9)
Score ≧ 6	41 (24.2)
Diseases, number (%)	
Hypertension	157 (92.9)
Dyslipidemia	135 (79.9)
Diabetes mellitus	27 (16.0)
Chronic kidney disease	31 (18.3)
Heart failure	20 (11.8)
Cerebral vascular disease	9 (5.3)
Cancer	10 (5.9)
Asthma	6 (3.6)
Chronic obstructive pulmonary disease	7 (4.1)

**Table 2:** The relationship between self-rated health and other demographic variables.

Variable	COR	95% CI	*p*	AOR	95% CI	*p*
Age (≧75 = 1, <75 = 0)	0.58	0.31–1.11	0.11	0.66	0.28–0.59	0.36
Sex (male = 1, female = 0)	0.77	0.42–1.44	0.42	0.84	0.34–2.08	0.71
Exercise (yes = 1, no = 0)	2.17	1.13–4.16	0.02	2.42	1.07–5.49	0.04
Balanced diet (yes = 1, no = 0)	2.43	1.06–5.53	0.04	2.58	0.83–8.01	0.1
Adequate sleep (yes = 1, no = 0)	5.22	2.22–12.27	<0.001	4.35	1.51–12.49	0.006
Work (yes = 1, no = 0)	1.98	1.05–3.75	0.04	2.59	1.08–6.22	0.03
Smoking (yes = 1, no = 0)	0.39	0.16–0.97	0.04	0.46	0.11–1.87	0.28
Regular drinking (yes = 1, no = 0)	1.06	0.54–2.05	0.87	1.49	0.54–4.18	0.44
High education (yes = 1, no = 0)	0.58	0.45–1.56	0.58	0.55	0.24–1.24	0.14
Social support (high = 1, low = 0)	0.43	0.56–3.83	0.43	0.62	0.17–2.31	0.47
SES (high = 1, low = 0)	3.38	1.49–7.69	0.004	6.67	2.13–20.88	0.001
Living alone (yes = 1, no = 0)	0.43	0.13–1.09	0.075	0.23	0.07–0.75	0.015
Social capital (high = 1, low = 0)	2.53	1.34–4.76	0.004	1.68	0.75–3.78	0.21
Health literacy (high = 1, low = 0)	1.17	0.63–2.17	0.62	1.12	0.51–2.46	0.78
CCI (Score ≧ 5 = 1, Score <5 = 0)	1.84	0.98–3.46	0.06	1.99	0.87–4.54	0.12
Help-seeking behavior						
Professional care only	1 (reference)	1 (reference)	1 (reference)	1 (reference)	1 (reference)	1 (reference)
Lay care use only	1.07	0.44–2.63	0.88	0.66	0.18–2.37	0.52
Both care use	1.38	0.67–2.86	0.38	2.39	1.02–6.29	0.046

Note. COR, crude odds ratio; AOR, adjusted odds ratio; *p*, *p*-value; SES, socioeconomic status; CCI, Charlson comorbidity index.

## Data Availability

The datasets used and/or analyzed during the current study may be obtained from the corresponding author upon reasonable request.
